# Inverse relationship between leukocyte telomere length attrition and blood mitochondrial DNA content loss over time

**DOI:** 10.18632/aging.103703

**Published:** 2020-07-23

**Authors:** Anthony Y.Y. Hsieh, Elana Kimmel, Neora Pick, Laura Sauvé, Jason Brophy, Fatima Kakkar, Ari Bitnun, Melanie C.M. Murray, Hélène C.F. Côté

**Affiliations:** 1 Department of Pathology and Laboratory Medicine, University of British Columbia, Vancouver V6T 2B5, British Columbia, Canada; 2 Centre for Blood Research, University of British Columbia, Vancouver V6T 1Z3, British Columbia, Canada; 3 Oak Tree Clinic, BC Women's Hospital, Vancouver V6H 3N1, British Columbia, Canada; 4 Women's Health Research Institute, Vancouver V6H 2N9, British Columbia, Canada; 5 Department of Medicine, Division of Infectious Diseases, University of British Columbia, Vancouver V5Z 1M9, British Columbia, Canada; 6 Department of Pediatrics, University of British Columbia, Vancouver V6H 0B3, British Columbia, Canada; 7 Department of Pediatrics, Children's Hospital of Eastern Ontario, University of Ottawa, Ottawa K1H 8L1, Ontario, Canada; 8 Department of Pediatrics, Centre Hospitalier Universtaire Sainte-Justine, Université de Montréal, Montréal H3T 1C5, Quebec, Canada; 9 Department of Pediatrics, Hospital for Sick Children, University of Toronto, Toronto M5G 1X8, Ontario, Canada

**Keywords:** telomere length, mtDNA content, HIV, immune aging, longitudinal study

## Abstract

Leukocyte telomere length (LTL) and whole blood mitochondrial DNA (WB mtDNA) content are aging markers impacted by chronic diseases such as human immunodeficiency virus (HIV) infection. We characterized the relationship between these two markers in 312 women ≥12 years of age living with HIV and 300 HIV-negative controls. We found no relationship between the two markers cross-sectionally. In multivariable models, age, ethnicity, HIV, and tobacco smoking were independently associated with shorter LTL, and the former three with lower WB mtDNA. Longitudinally, among a subgroup of 228 HIV participants and 68 HIV-negative controls with ≥2 biospecimens ≥1 year apart, an inverted pattern was observed between the rates of change in LTL and WB mtDNA content per year, whereby faster decline of one was associated with the preservation of the other. Furthermore, if HIV viral control was not maintained between visits, increased rates of both LTL attrition and WB mtDNA loss were observed. We describe a novel relationship between two established aging markers, whereby rates of change in LTL and WB mtDNA are inversely related. Our findings highlight the importance of maintaining HIV viral control, the complementary longitudinal relationship between the two markers, and the need to consider both in aging studies.

## INTRODUCTION

Telomeres are nucleoprotein complexes that cap chromosomes and, in most cells, shorten with each division. Leukocyte telomere length (LTL) is associated with age [1], and the rate of LTL attrition is more rapid during childhood (100-1000 bp/year) [2] than adulthood (20-100 bp/year) [3]. Biological variability in LTL is attributed to genetics [4], sex [5], ethnicity [6], and other determinants of health including diet [7], exercise [8], and substance use such as tobacco smoking [9, 10] and illicit drugs [11]. Shorter telomere length (TL) in leukocytes or peripheral blood mononuclear cells is also associated with human immunodeficiency virus (HIV) [12–15], diabetes [16], Alzheimer’s disease [17], and certain cancers [18, 19]. Among older individuals, TL is particularly predictive of cardiovascular disease [20–22] and mortality [23].

Mitochondria harbor multiple copies of mitochondrial DNA (mtDNA) per cell, which replicate independently from nuclear DNA. Changes in mtDNA quality or quantity, as well as decline in mitochondrial function, are implicated in biological aging [24] and are primarily linked to metabolic and mitochondrial disorders. Similarities also exist between whole blood (WB) mtDNA content per cell and LTL, whereby many diseases or conditions associated with shorter LTL are also associated with differences in mtDNA content. For example, decreased mtDNA content in various tissues is associated with HIV [25–27], diabetes [28], Alzheimer’s disease [29], various cancers [30, 31], and cardiovascular disease [32]. Tobacco smoking is also associated with altered mtDNA content in lung and buccal cells, though reports are inconsistent [33, 34]. Compared to LTL, which is consistently reported to decline with age, WB mtDNA content is not as robust a marker of aging, with studies having reported negative [24], positive [35, 36], non-linear [37, 38], or no relationships [39] between mtDNA content and age.

Several proposals have arisen connecting these two markers of cellular aging mechanistically. Short telomeres trigger p53 activation, which itself is associated with mitochondrial biogenesis [40]. It is also well recognized that telomerase translocates to and protects mitochondria in conditions of oxidative stress [41, 42]. Despite this, the relationship between LTL and WB mtDNA content is unclear based on cross-sectional studies [43–46] and has not yet been investigated longitudinally.

In the context of HIV, even people successfully treated with combination antiretroviral therapy (cART) have an accelerated aging phenotype compared to their HIV-negative peers [47]; they also have shorter LTL and abnormal mtDNA content. Despite a growing body of literature linking aging, HIV, and other diseases to either shorter LTL or altered WB mtDNA content individually, there is a paucity of research considering the two markers together, describing the relationship between the two, especially longitudinally. Given that HIV may provide a human model of biological aging, our goal was to characterize the relationship between LTL and WB mtDNA content in a cohort of girls and women living with and without HIV, both cross-sectionally and longitudinally.

## RESULTS

### Study sample

Characteristics of the study participants are shown in Table 1. Cross-sectional analyses included 312 women living with HIV (WLWH) and 300 HIV-negative participants. WLWH were slightly younger, with a median (range) age of 41 (14-69) *vs.* 44 (15-78) years (P=0.042). WLWH were also more likely to be African/Caribbean/Black (ACB), current tobacco smokers, current prescribed opioid users, have past or present HBV or HCV infections, and less likely to be White, Asian, current drinkers, have a household income >15000 CAD/year, and have any college education compared to HIV-negative controls. Body mass index and cannabis use did not differ between groups. Among WLWH, 70% of participants had an undetectable HIV plasma viral load (pVL <50 HIV RNA copies/ml) and 82% were on cART at visit. Among all participants, the median[interquartile range] LTL and WB mtDNA content measurements were 7.2[6.5-7.9] and 105[81-139], respectively.

**Table 1:** Study sample characteristics.

	**Cross-Sectional Sample (N=612)**			**Longitudinal Sample (N=296)**		
	**HIV+ (N=312)**	**HIV- (N=300)**	**P-value**	**HIV+ (N=228)**	**HIV- (N=68)**	**P-value**
**Age,** years	41 [31,50](14,69)	44 [31,55](15,78)	**0.042**	38 [29,46](12,67)	36 [27,48](12,73)	0.859
**Time between visits,** years				4 [2,6] (1,8)	3 [2,5] (1,7)	**< 0.001**
**BMI,** kg/m^2^ (N=603)	24.9 [21.4,30.0] (15.0,48.6)	24.6 [21.4,29.7] (14.0,52.9)	0.608	24.0 [21.3,28.8] (16.0,46.6)	23.5 [20.5,28.7] (15.3,42.3)	0.526
**Ethnicity**			**< 0.001**			
White	131 (42)	153 (51)		94 (41)	37 (54)	**0.003**
African/Black/Caribbean	72 (23)	19 (6)		51 (22)	3 (4)	
Indigenous	78 (25)	79 (26)		58 (25)	16 (24)	
Asian	8 (3)	28 (9)		8 (4)	7 (10)	
Other^a^	23 (7)	21 (7)		17 (7)	5 (7)	
**Household income >$15000 CAD/year** (N=553)	127 (48)	170 (59)	**0.009**	95 (48)	40 (67)	**0.011**
**Highest level of education** (N=552) Any College	114 (43)	200 (69)	**< 0.001**	93 (47)	44 (73)	**0.005**
High School Graduate	54 (21)	30 (10)		38 (19)	6 (10)	
Some High School	80 (30)	53 (18)		56 (28)	9 (15)	
Grade School	15 (6)	6 (2)		11 (6)	1 (2)	
						
**Cigarette smoking^b^**						
Current	122 (39)	85 (28)	**0.010**	85 (37)	13 (19)	**0.002**
Past	69 (22)	67 (22)		54 (24)	12 (18)	
Never	121 (39)	148 (49)		89 (39)	43 (63)	
**Cannabis^b^**						
Current	77 (25)	72 (24)	0.853	59 (26)	15 (22)	0.664
Past	67 (21)	60 (20)		49 (21)	13 (19)	
Never	168 (54)	168 (56)		120 (53)	40 (59)	
**Alcohol^b^**						
Current	150 (48)	199 (66)	**< 0.001**	113 (50)	44 (65)	**0.039**
Past	87 (28)	61 (20)		63 (28)	17 (25)	
Never	75 (24)	40 (13)		52 (23)	7 (10)	
**Current any opioid use^b^**	69 (22) 62 (20)	32 (11) 25 (8)	**< 0.001**	51 (22) 47 (21)	3 (4) 1 (1)	**< 0.001**
Prescribed opioid use			**< 0.001**			**< 0.001**
Heroin use	17 (5)	13 (4)	0.523	15 (7)	0 (0)	**0.030**
**HIV Detectable pVL at visit,** >50 copies/ml^**b,c**^	95 (30)			63 (28)		
**HIV Peak pVL >100000** copies/ml (N=248)	111 (45)			87 (48)		
**CD4 count at visit,** cells/μL^**b**^	540 [348,733] (10,2380)			500 [348,683] (14,1370)		
**CD4 nadir,** cells/μL (N=260)	221 [130,350] (1,1110)			216 [118,330] (1,900)		
**ART-naïve** (N=278)	10 (4)			11 (5)		
**On cART at visit^b^**	255 (82)			195 (86)		
**HBV ever infected** (N=395)	29 (13)	2 (1)	**< 0.001**	9 (26)	0 (0)	**0.011**
**HCV ever infected**	109 (35)	45 (15)	**< 0.001**	79 (35)	9 (13)	**< 0.001**
**Platelet Count,** 10^9^/L (N=429)	227 [178,274] (40,663)	235 [213,281] (82,464)	**0.005**	233 [184,274] (45,489)	272 [217,277] (172,316)	0.240
**Relative LTL**	7.1 [6.4,7.8] (4.8,11.5)	7.4 [6.7,8.1] (4.8,11.3)	**0.004**	7.0 [6.3,7.8] (4.7,10.5)	7.5 [6.9,8.4] (5.1,10.5)	**< 0.001**
**ΔLTL/year**			**0.004**	0.01 [-0.10,0.11] (-0.94,1.80)	-0.05 [-0.26,0.09] (-1.18,0.74)	**0.020**
**WB mtDNA content**	101 [78,131] (4,265)	112 [85,146] (4,379)		143 [109,171] (4,277)	129 [96,154] (4,231)	**0.025**
**WB ΔmtDNA content/year**				-10 [-15,-1] (-76,77)	-7 [-16,8] (-32,37)	**0.013**

Data are number (%) of individuals or median [interquartile range] (range). Number of participants with available data indicated beside variables with incomplete data. P-values indicate Mann-Whitney U or Chi-Squared Tests depending on type of variable.

Abbreviations: ART, antiretroviral therapy; BMI, body mass index; cART, combination antiretroviral therapy; CAD, Canadian dollars; HBV, hepatitis B virus; HCV, hepatitis C virus; LTL, leukocyte telomere length; mtDNA, mitochondrial DNA; pVL, plasma viral load; WB, whole blood.

^a^ a combination of South Asian, Hispanic, or other

^b^ at last visit for longitudinal sample

^c^ 22 lost HIV viral control and 41 participants gained HIV viral control between longitudinal visits

### Cross-sectional LTL and WB mtDNA content decline with age but are not inter-related

**Figure 1:**
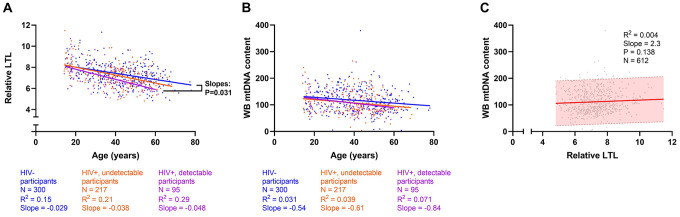
**Cross-sectional univariate associations between leukocyte telomere length (LTL), whole blood mitochondrial DNA (WB mtDNA) content, and age.** Among 612 participants, (**A**) LTL declines with age (R^2^=0.17, Slope=-0.03, Pearson’s P<0.0001); as does (**B**) WB mtDNA content (R^2^=0.03, Slope=-0.57, P<0.0001). Both measures also decline within each HIV subgroup (all p<0.01). Linear regressions of HIV-, HIV+ undetectable plasma viral load (pVL), and HIV+ with detectable pVL participants are shown in blue, orange, and magenta, respectively. Differences between slopes were tested and showed that (**A**) LTL declines faster among HIV+ detectable pVL participants than in HIV- controls. (**C**) No detectable relationship exists between LTL and WB mtDNA content (P=0.138). The shaded area indicates the 95% prediction interval. Coefficients of determination (R^2^) are shown.

WLWH had shorter LTL (median 7.1 *vs.*7.4) and lower WB mtDNA content (median 101 *vs.* 112) than HIV-negative controls before (Table 1, P≤0.004) and after adjusting for age (P≤0.002). Among all participants, relative LTL declined with age (Figure 1A), with an average loss of approximately 33 bp of telomeric DNA per year (R^2^=0.17, P<0.0001). LTL was modulated by HIV status, whereby the decline in LTL with age was faster among WLWH with detectable pVL compared to HIV-negative controls. WB mtDNA also declined with age (Figure 1B), although with a weaker relationship (R^2^=0.03, P<0.001) that was not modulated by HIV. Despite the fact that both markers declined with age, they were not associated with one another (Figure 1C) (R^2^=0.004, P=0.14).

### Cross-sectional predictors of LTL

**Figure 2:**
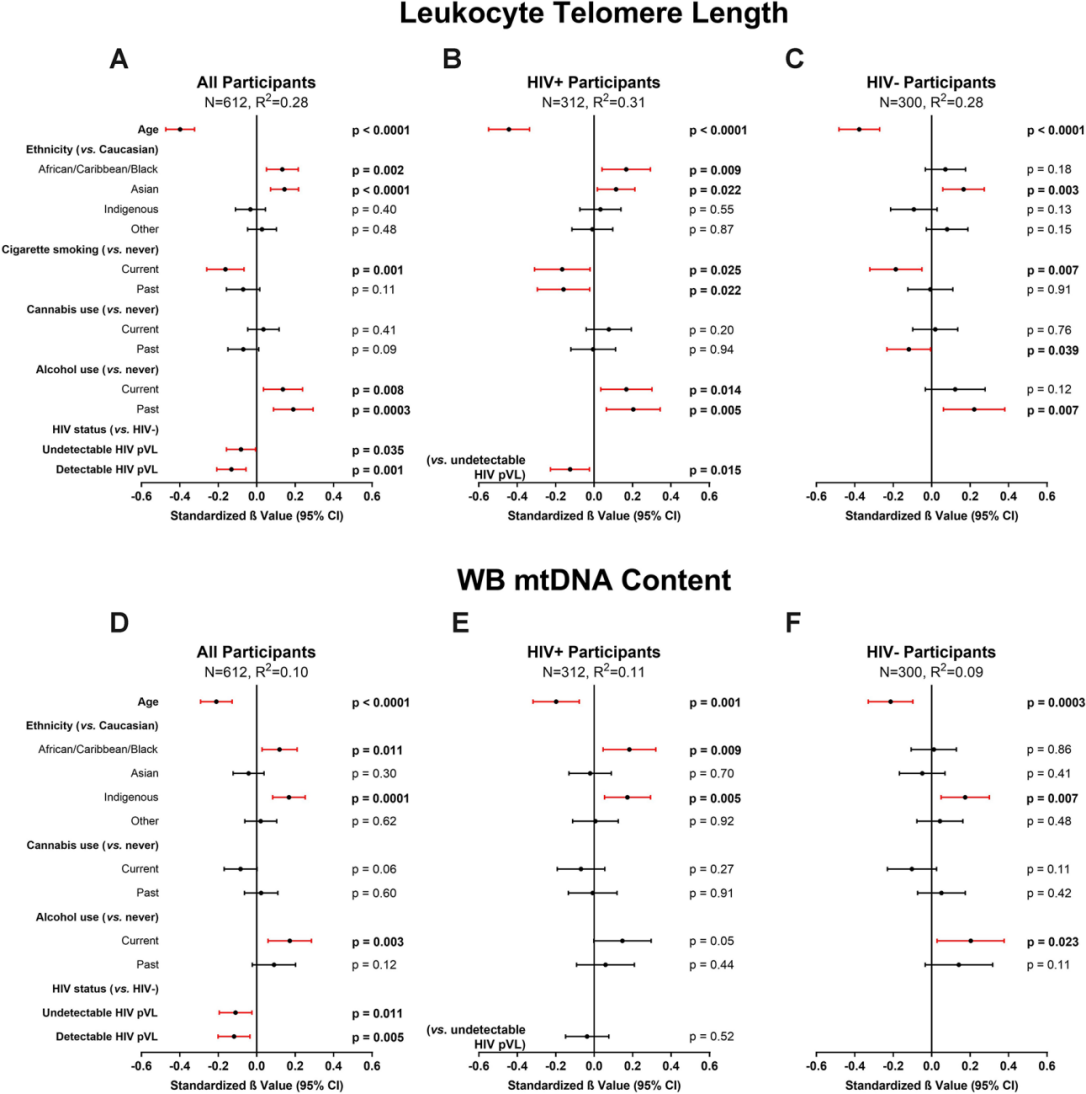
**Multivariable modelling of cross-sectional leukocyte telomere length (LTL) and whole blood mitochondrial DNA (WB mtDNA) content.** Final selected multivariable linear regression models of cross-sectional LTL (variance inflation factor (VIF) ≤2.1) in (**A**) all, (**B**) HIV+, and (**C**) HIV- participants, and WB mtDNA content (VIF≤1.3) in (**D**) all, (**E**) HIV+, and (**F**) HIV- participants. Final models among all participants were selected automatically by minimizing Akaike’s Information Criterion (AIC). Statistical significance depicted by red confidence intervals; negative standardized β values indicate associations with either shorter LTL or lower WB mtDNA content and vice versa. Coefficients of determination (R^2^) are shown for each model. The models show that after adjusting for age, ethnicity and substance use, HIV infection is independently associated with shorter LTL and lower WB mtDNA content. Detectable HIV viremia was associated with shorter LTL but not WB mtDNA content.

Among all participants, the final multivariable model for LTL (N=612, R^2^=0.28) indicated that HIV infection (either with detectable or undetectable pVL), current tobacco smoking, and older age were independently associated with shorter LTL. In contrast, alcohol use and ACB or Asian ethnicities were associated with longer LTL (Figure 2A). Cannabis use showed no effect on LTL. In a subgroup of WLWH (Figure 2B), detectable HIV pVL was also independently associated with shorter LTL compared to undetectable HIV pVL (N=312, R^2^=0.31), in addition to the above factors. Among HIV-negative participants, current smoking remained associated (N=300, R^2^=0.28) with shorter LTL, but current alcohol did not (Figure 2C). Models showing non-standardized effect sizes are shown in supplement (Supplementary Figure 2). Although substance use was considered when building our model, to ensure the validity of our findings in the general population, we performed subgroup analyses among participants who never used tobacco, cannabis, or opioids (Supplementary Figure 3A–3C). Similarly, informed by past studies that showed effects on LTL by chronic co-infections [12], we analyzed participants with no history of either HCV or HBV infection (Supplementary Figure 3D). All models showed essentially the same effects as the main model, and in the smaller never smoker and never HBV/HCV-coinfected groups, the effect size of undetectable HIV infection was similar, but the confidence interval became broader and significance was lost. The effect of detectable HIV infection remained in all subgroup models. Taken together, these secondary models confirmed the robustness of the effects observed in our main model.

### Cross-sectional predictors of WB mtDNA content

The final multivariable model for WB mtDNA content (N=612, R^2^=0.10) indicated that HIV infection and older age were independently associated with decreased WB mtDNA content, while ACB and Indigenous ethnicities, as well as current alcohol use, showed an association with increased WB mtDNA content (Figure 2D). Contrary to the LTL models, no difference was detected between participants with detectable or undetectable HIV pVL (Figure 2E). Once again, we carried out subgroup analyses as above and found that older age remained in all models while the effect of HIV was detected sporadically, likely due to lower power and wider confidence intervals (Supplementary Figure 4).

Sensitivity models were constructed including platelet count data that were available for a subset of study participants. Here, higher platelet count showed an association with higher WB mtDNA content (Supplementary Figure 5A–5C) and the relationship between older age and decreased WB mtDNA content remained, but the effect of HIV was lost. However, in these models, the sample size of the HIV-negative group was considerably reduced (N=121 *vs.* N=300).

### Longitudinal changes in LTL and mtDNA are inversely related

Having determined that both age and HIV affect our two markers cross-sectionally, we then examined the dynamics of the two markers over time, as well as their longitudinal relationship. Of the 612 participants studied cross-sectionally, 228 WLWH and 68 HIV-negative participants had at least one visit ≥1 year earlier and were included in the longitudinal analyses. The median time between longitudinal visits was 4 and 3 years for WLWH and HIV-negative participants, respectively. Characteristics of the longitudinal subset were similar to whole group (Table 1) with the exception that age was similar between groups, and baseline mtDNA content was higher in WLWH than HIV-negative controls. Medians [interquartile range] of 4 [2, 6], and 3 [2, 5] years elapsed between longitudinal visits in WLWH and HIV-negative controls, respectively.

**Figure 3:**
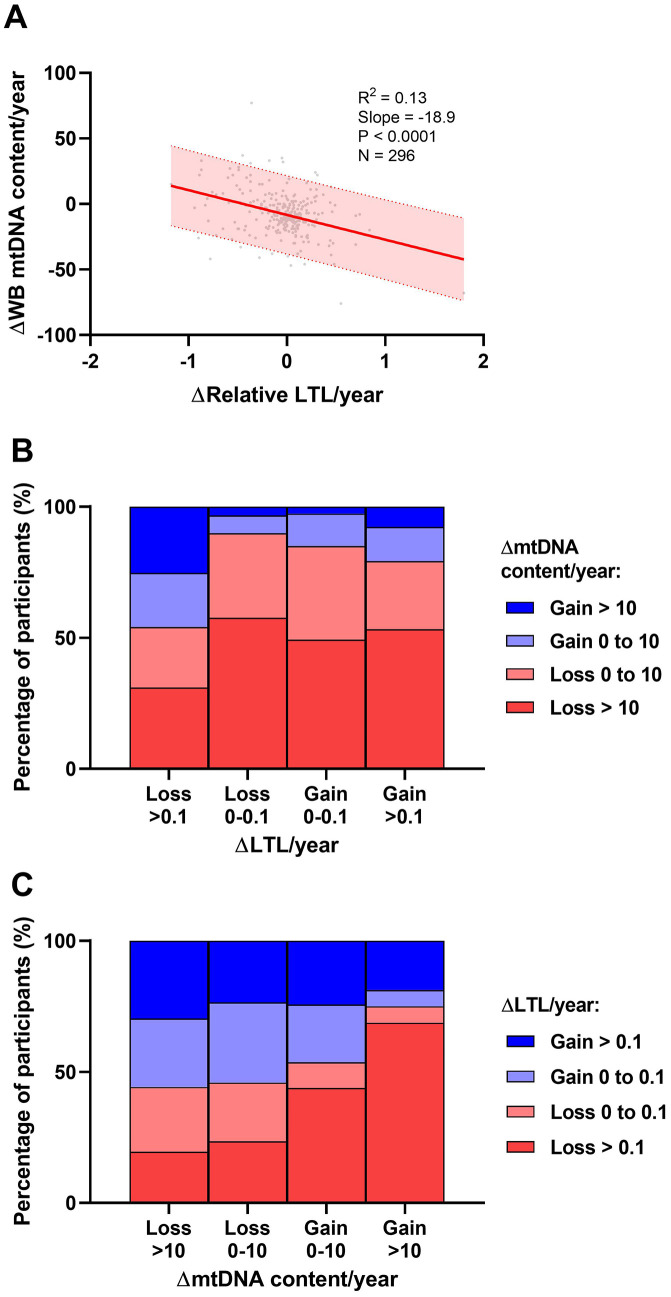
**Univariate associations between longitudinal rates of change in leukocyte telomere length (LTL) and whole blood mitochondrial DNA (WB mtDNA) content.** (**A**) Data show an inverse relationship between ΔLTL/year and WB ΔmtDNA content/year. Shaded area represents the 95% prediction interval. Coefficient of determination (R^2^) and Pearson's P-value shown. (**B**) Participants were categorized based on a small (<0.1) or large (>0.1) loss or gain of ΔLTL/year and further stratified according to a small (<10) or large (>10) loss or gain of WB ΔmtDNA content/year. These data show that participants who lost LTL fastest were more likely to preserve or gain WB mtDNA content. (**C**) Similarly, participants were categorized based on WB ΔmtDNA content/year and stratified according to ΔLTL/year. The data show that participants who lost WB mtDNA fastest were more likely to preserve LTL and vice versa.

A significant negative relationship was observed between ΔLTL/year and WB ΔmtDNA content/year (R^2^=0.13, P<0.001) (Figure 3A). Overall, participants who lost LTL the fastest were also most likely to have gained mtDNA over the same period (Figure 3B) and vice versa whereby those who lost mtDNA the fastest were more likely to have gained LTL (Figure 3C).

### Predictors of longitudinal change in LTL

**Figure 4:**
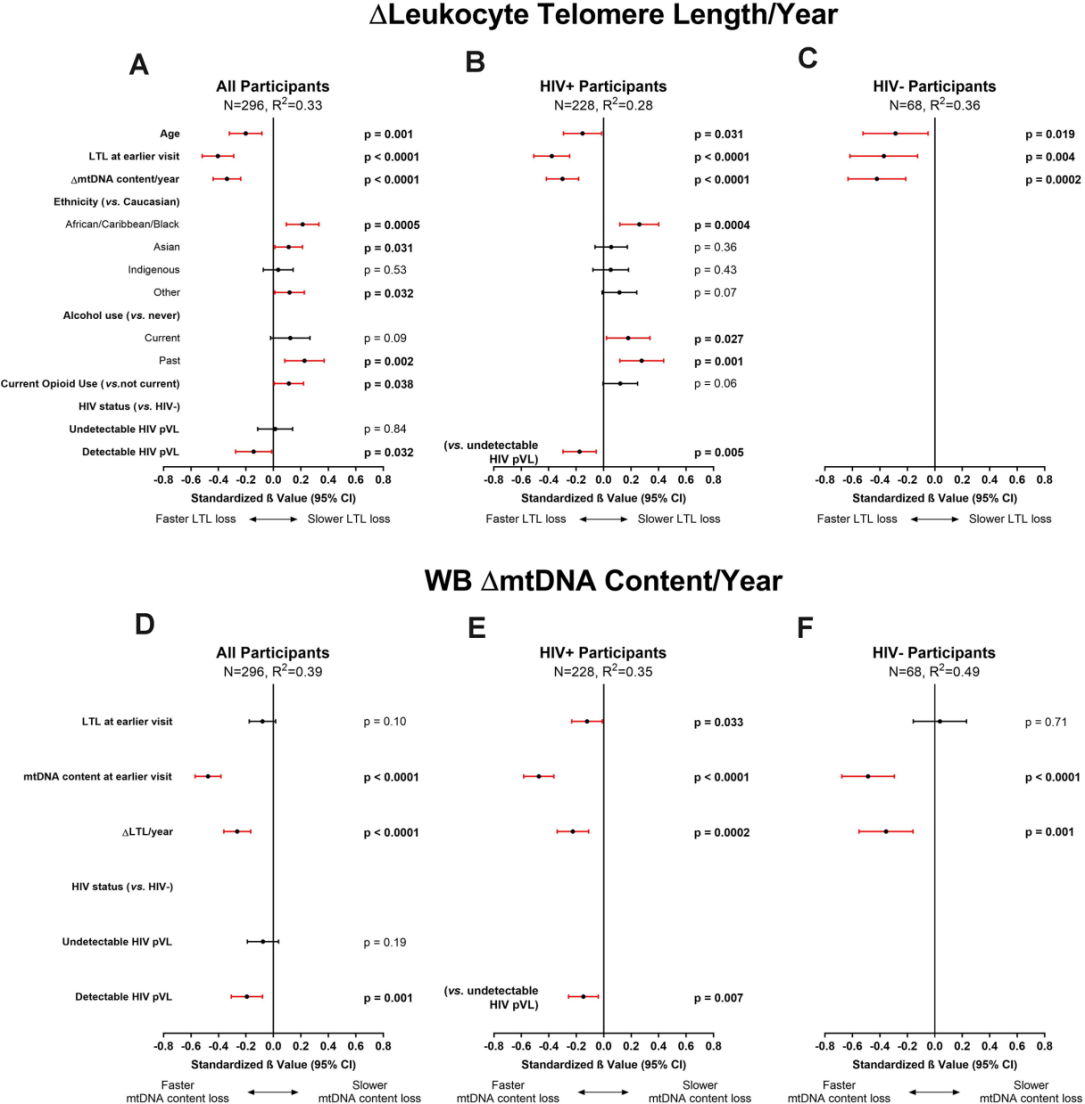
**Multivariable modelling of longitudinal rates of change in leukocyte telomere length (LTL) and whole blood mitochondrial DNA (WB mtDNA) content.** Final selected multivariable linear regression models of longitudinal ΔLTL/year (variance inflation factor (VIF) ≤1.5) in (**A**) all, (**B**) HIV+, and (**C**) HIV- participants, and WB ΔmtDNA content/year (VIF≤1.2) in (**D**) all, (**E**) HIV+, and (**F**) HIV- participants. Final models among all participants were selected automatically by minimizing Akaike’s Information Criterion (AIC). Statistical significance depicted by red confidence intervals; negative standardized β values indicate associations with either faster LTL loss or faster WB mtDNA content loss and vice versa. Coefficients of determination (R^2^) are shown for each model. The ΔLTL/year models show that after adjusting for age and LTL at baseline, ethnicity, and substance use, a slower loss of WB mtDNA/year is significantly independently associated with faster LTL attrition. Similarly, the models for WB ΔmtDNA content/year show that after adjusting for LTL and WB mtDNA at baseline, a slower rate of LTL attrition is independently associated with faster loss of WB mtDNA content. Detectable HIV viremia was associated with faster decline in both markers.

In the univariate analysis, overall relative LTL shortened by 0.036 units/year among all participants. This represents a loss of approximately 36 bp/year, in close agreement with the population attrition rate estimate (33 bp/year) seen in the cross-sectional analysis (Figure 1A). These univariate investigations were validated by multivariable modelling (N=296, R^2^=0.33), in which we observed that a slower decrease in WB mtDNA content/year was a strong predictor of faster LTL loss (Figure 4A), as was having longer LTL at baseline. Older age at baseline was also associated with faster LTL loss while past alcohol use, current opioid use, as well as ACB and Asian ethnicities were associated with slower loss of LTL (Figure 4A), reminiscent of the cross-sectional analysis. In contrast to the cross-sectional finding, tobacco smoking and undetectable HIV did not show an effect on LTL attrition rate. However, among WLWH, LTL was lost faster in those with a detectable HIV pVL at last visit (Figure 4B). Among HIV-negative participants (Figure 4C), only age, baseline LTL and WB ΔmtDNA content were included due to the reduced sample, and they all showed similar effects as those seen in the all-participants model. Models showing non-standardized effect sizes are shown in supplement (Supplementary Figure 6). In all subgroup analyses that excluded substance users or co-infections (Supplementary Figure 7), slower loss of WB mtDNA content/year and longer baseline LTL remained independently associated with faster LTL attrition. Similar to the cross-sectional subgroup models, the effect of HIV lost significance in some models, likely due to reduced power.

Taken together, our data show that the main predictors of faster LTL attrition were having longer LTL at baseline and a slower WB mtDNA content decline over time. Our data also point toward faster LTL attrition among those who have a detectable HIV pVL while those who maintain viral control showed rates of LTL loss essentially identical to HIV-negative individuals.

### Predictors of longitudinal change in WB mtDNA content

In WLWH and controls, WB mtDNA content decreased by medians of 10 and 7 copies of mtDNA per copy of nDNA/year, respectively (Table 1). Similar to what was observed for ΔLTL, in the final model of WB ΔmtDNA content (N=296, R^2^=0.39), detectable HIV infection, higher WB mtDNA content at baseline, and slower LTL attrition rate were each independently associated with a faster decline in WB mtDNA content (Figure 4D). Among WLWH (Figure 4E) and HIV-negative controls (Figure 4F), the same associations were seen. Furthermore, compared to WLWH with undetectable HIV pVL, having a detectable HIV pVL at last visit was associated with faster WB mtDNA content decline (Figure 4E). Subgroup analyses were highly similar to the all-participants model (Supplementary Figure 8) and the effect of viremia persisted in all models. Adding platelet counts to the WB ΔmtDNA content models had no effect, and platelet count itself showed no association with WB ΔmtDNA content (Supplementary Figure 9).

Overall, as seen for the change in LTL, the strongest predictors of faster decrease in WB mtDNA content were higher WB mtDNA content at baseline and slower LTL loss. In addition, having a detectable HIV pVL accelerated mtDNA loss.

## DISCUSSION

This is the first human cohort study to incorporate both LTL and WB mtDNA content measures and their rates of change over time, adjusting for relevant covariates. Our primary finding is that the rates of change in the two measures are inversely associated, meaning that while both decline with age, a faster decline in one marker is observed if the decline in the other is slower, and vice versa. We further show the negative impact of uncontrolled HIV viremia on both LTL and WB mtDNA content loss longitudinally. Given that both markers were measured using the same technique (monochrome multiplex quantitative polymerase chain reaction, MMqPCR) on the same cohort specimens, this study was uniquely suited to characterize the relative effect of predictors, as well as the influence of one marker on the other. These data highlight the importance of investigating both LTL and WB mtDNA content as complementary and non-independent markers of aging.

The inverse relationship between LTL and mtDNA content attrition rates, seen in both the ΔLTL/year and the ΔmtDNA content/year models, is a novel finding and sheds light on previously proposed mechanisms that implicate a relationship between telomere and mitochondrial biology. This effect is consistent with the dual functions of telomerase. While the classical function of telomerase is to maintain telomeres, *in vitro* research has demonstrated that under oxidative stress conditions, the enzymatic subunit of telomerase translocates to the mitochondria where it exerts a protective function against oxidative damage but appears to neglect telomere maintenance [41, 42]. Telomerase localization to mitochondria has also been demonstrated *in vivo* in the neurons of both mice and humans [48, 49]. A similar phenomenon may be occurring in leukocytes when mitochondria require protection, potentially resulting in mitigated mtDNA loss at the cost of higher LTL attrition rate, as seen here. If so, this would suggest that upon oxidative stress challenge, telomerase confers a necessary and immediate protection to the mitochondria at the expense of the long-term benefit of telomere maintenance. Furthermore, inter-related mechanisms of mitochondrial metabolism, telomerase-associated DNA damage sensing, and ribosome production, previously described as the mitochondrion/telomere nucleoprotein complex/ribosome (MTR) postulate [50], have been implicated in cellular aging, in which disruption of any one mechanism may lead to consequences throughout all three systems. The potential mechanistic relationships between LTL, WB mtDNA content, and other previously established markers of aging and/or senescence should also be explored. For example, HIV has been linked with changes in expression of cyclin-dependent kinase inhibitor 2A (CDKN2A) [51] and DNA methylation levels [52]. As yet, no direct evidence links the relationship between LTL and mtDNA content attrition rates to any of the mechanisms described above. Future longitudinal studies in an independent cohort with a longer interval between measures are needed to confirm this relationship and determine if this apparent “yin and yang” effect persists over longer periods of time.

The rate of LTL attrition observed agrees with previous research [3] and is consistent between our cross-sectional and longitudinal data. Although WB mtDNA content also declined with age, both univariable and multivariable analyses explained a smaller percentage of the total variance compared to that of LTL models. Given that the variability of each of the two assays is comparable [53, 54], this suggests that either WB mtDNA content is intrinsically more stochastic, or that the mtDNA analyses presented here missed key explanatory variables. Such yet unidentified variables may partially explain the inconsistency in previous attempts by researchers to establish WB mtDNA content as a robust marker of aging. A potential confounder that we addressed in WB mtDNA content models was platelets, which may lead to overestimation of WB mtDNA content. Two studies have shown that a very large increase in platelet mtDNA contribution would be necessary to meaningfully affect WB mtDNA content measurement [55, 56], and others found no relationship between platelet count and WB mtDNA content [26, 57]. Based on this and our own unpublished data in a non-HIV sample, we did not initially intend to include platelet count in our analyses. However, a recent study highlighted the importance of platelet count in studies investigating the effect of HIV on WB mtDNA content [27]. Therefore, to increase the rigor of our analysis, we analyzed a subset of our participants for whom platelet count data were available to investigate the role of platelets in WB mtDNA content and its change over time. We found that platelet count had no effect on our longitudinal models. Despite a modest reduction in power, the effects of HIV on faster WB mtDNA attrition, as well as the inverse relationship between the rates of change in LTL and WB mtDNA content over time, persisted. However, while platelet count affected WB mtDNA content cross-sectionally, its inclusion resulted in a substantial reduction in sample size and the effect of HIV lost statistical significance. It remains unclear whether the well-established relationship between HIV and decreased WB mtDNA content is partially driven by thrombocytopenia in people living with HIV.

We could not detect any significant cross-sectional relationship between LTL and WB mtDNA content, whether univariately or multivariately, in contrast to some studies in healthy adults that reported modest correlations (R^2^=0.15, and R^2^=0.03) [36, 44]. The reason for this is unclear and may be related to differences in the assays used and the range they yield. There could also be a bias toward not reporting negative findings (i.e. no correlation) by other studies. Furthermore, past studies did not examine this relationship while adjusting for covariates and confounders.

HIV infection and uncontrolled viremia have been previously associated with both shorter LTL [12] and lower mtDNA content [26, 27]. Based on non-standardized effect sizes, our study demonstrates that the independent effects on LTL by HIV infection, uncontrolled HIV viremia, and current tobacco smoking are each similar to approximately one decade of chronological aging. Furthermore, we show that the decline of both LTL and mtDNA content with age is accelerated in participants with uncontrolled HIV viremia, emphasizing the importance of maintaining HIV viral control. This is supported by model estimates showing that having uncontrolled viremia increases the LTL loss per year of aging from approximately 30 bp to more than 100 bp.

Previous research has shown early and rapid LTL decline following HIV acquisition [14, 15], which may result from an initial HIV-induced immune activation leading to rearrangement of leukocyte subsets. It is possible that a similar phenomenon is occurring here, in which immune cell proliferation triggered by actively replicating HIV results in faster apparent attrition of both LTL and WB mtDNA content. A recent study demonstrated faster mtDNA content attrition among WLWH who were approximately 50 years old compared to controls [27]. However, more research with longitudinal samples spanning longer periods of time is necessary to determine whether this effect is transient or carries long-term consequences. Indeed, these findings are in keeping with the model of HIV-mediated accelerated aging and may explain the link between HIV and aging comorbidities. While HIV represents a valuable model of accelerated human aging, other chronic and proinflammatory diseases such as diabetes, rheumatoid arthritis, and other chronic/latent viral infections associated with aging comorbidities may similarly modulate LTL and mtDNA content.

In support of previous research, we show that tobacco smoking is robustly independently associated with shorter LTL. In most subgroup analyses, this effect exists only among current smokers, suggesting that smoking cessation confers a benefit to LTL, as reported by others [10]. However, it is unclear why the benefit of smoking cessation was not detected among WLWH. It is possible that time since smoking cessation differed between groups. Alternatively, the mechanism by which LTL recovers after smoking cessation may be adversely affected by the presence of HIV. Smoking was also not associated with LTL attrition rate, likely because the time elapsed between longitudinal visits was insufficient for the effect of smoking to reach a detectable size.

In contrast, alcohol use was associated with longer LTL, slower LTL attrition, and increased WB mtDNA content. While this may seem to contradict some research reporting shorter LTL in people who abuse alcohol [58], other studies are less clear as to the relationship between LTL and moderate alcohol consumption [21, 59]. Our definition for this variable was any alcohol use regardless of quantity, which does not imply abuse and would also include low to moderate users. Moreover, unlike tobacco smoking or opioid use, alcohol use is lower among our WLWH. The apparent association between alcohol use and both longer LTL and increased WB mtDNA content may therefore be the result of nested effects with unknown factors more prevalent among the HIV-negative group, and not fully addressed by the covariates considered herein. It is also possible that we detected a spurious finding, as the association with alcohol disappeared in some subgroup analyses. Nevertheless, the associations between alcohol and the markers studied here are of interest and should be reproduced in an independent cohort, along with a more granular analysis of alcohol consumption, with respect to frequency and quantity.

While differences in LTL dynamics between men and women exist, this study includes only female participants. As such, we are not able to comment on whether the same predictors of LTL and WB mtDNA content would be seen in men. Furthermore, this analysis also does not consider the intensity and frequency of substance use. The role of sex and substance use should therefore be further examined.

In summary, we describe a novel relationship between longitudinal LTL and WB mtDNA dynamics, whereby better preservation of LTL appears to occur at the cost of mtDNA loss and vice versa. We also validate the cross-sectional predictors of LTL and WB mtDNA content that have been previously shown. In addition, we demonstrate that for women living with HIV, loss of viral control exacerbates both LTL and WB mtDNA content attrition and may contribute to biological aging. Taken together, our findings provide further evidence supporting the importance of consistently maintaining HIV viral control. Given that the effects of HIV viremia are believed to be largely related to chronic inflammation, it is possible that other proinflammatory conditions would similarly exacerbate cellular aging through related mechanisms. Our findings further imply that future longitudinal studies of either LTL or WB mtDNA content as a biomarker should also consider the other given their strong relationship.

## MATERIALS AND METHODS

### Study sample

Study participants included all non-pregnant women and girls ≥12 years old living with or without HIV enrolled in the Children and Women: AntiRetrovirals and Markers of Aging (CARMA) cohort between December 2008 and August 2017. Enrolment and blood collection of WLWH took place during routine clinical visits at four sites across Canada. HIV-negative controls were purposely recruited to have similar sociodemographic characteristics as the HIV group. Written informed consent was provided by adult participants, and assent from pediatric participants was obtained with parent/legal guardian consent. This study was approved by the University of British Columbia Research Ethics Board at the Children’s and Women’s Hospital (H08-02018). Further details on CARMA enrolment have been previously described [12], and more information can be found in Supplementary methods.

Study participants total 328 WLWH and 318 HIV-negative women and girls. Of these, one participant was excluded because no blood was collected and 33 were excluded because of missing demographic or clinical data that were deemed essential *a priori* (Supplementary Figure 1). These variables included age, ethnicity, tobacco smoking, cannabis use, alcohol use, opioid use, HCV infection ever, and HIV status for all participants, as well as current HIV pVL, and CD4 count at visit for WLWH. Other data considered included BMI, household income, highest education level completed, HCV viremia, highest HIV pVL ever recorded (peak pVL), lowest CD4 count recorded, and platelet count (Supplementary Table 1). For participants with ≥2 study visits at least one year apart, the last visit was used for cross-sectional analyses, and the earliest (or baseline) and latest visits were used for longitudinal analyses.

In total, cross-sectional analyses included 312 WLWH and 300 HIV-negative participants. Longitudinal analyses included 228 WLWH and 68 HIV-negative participants.

### Biospecimen collection and qPCR

WB was collected and stored at -80°C until genomic DNA was extracted from 100 μl of WB using the QIAamp DNA Mini Kit on the QIAcube (Qiagen, Hilden, Germany) according to the manufacturer’s blood and body fluid protocol. DNA was eluted in 100 μl of Buffer AE which contains 0.5 mM EDTA and 10 mM Tris-Cl, pH 9.0, and stored at -80^o^C until assayed.

Relative LTL and mtDNA content were measured in duplicate by MMqPCR as previously described [53, 54]. TL was defined as the ratio between telomeric DNA quantity and the copy number of a single-copy nuclear gene, albumin (*ALB*). MtDNA content was defined as the ratio of mitochondrial genome copy number, measured using a segment of the mitochondrial displacement loop, to *ALB* copy number. The TL assay was calibrated using fluorescent *in situ* hybridization such that each unit of relative TL approximates 1 kb of telomeric DNA, whereas the mtDNA content assay provides the mtDNA to nuclear DNA ratio. Both LTL and WB mtDNA content were assayed in whole blood, producing measurements for the average leukocyte. In a previous publication by our group, the intra- and inter-assay variabilities of the TL assay were measured among several operators to be 4.2-6.2% and 3.2-4.9%, respectively [53]. Similarly, the intra- and inter-assay variabilities of the mtDNA content assay were determined to be 4.3-7.9% and 2.9-9.2%, respectively [54].

### Statistical analyses

To evaluate the relationship between potential explanatory variables and our measures of interest, namely LTL and mtDNA content and the change in these over time, possible associations were first explored with univariate analyses using Pearson’s or Spearman’s correlation, one-way analysis of variance (ANOVA), Kruskal-Wallis, Student’s t-, or Mann-Whitney U tests. Variables that were important in univariate analysis (P<0.15) were candidates for inclusion in multivariable models. The final models for each measure of interest included all essential variables as described above and were constructed in a stepwise manner, by minimizing the Akaike information criterion (AIC). While possible interactions were explored, none offered substantial model improvement. Chi-Square contingency tables, Kruskal-Wallis tests, and Spearman’s correlation were used to detect collinearity among variables, and variance inflation factors (VIFs) were calculated to characterize the influence of collinearity in the models. To further test the robustness of final models, subgroup analyses (such as among a specific ethnicity or HIV group) and sensitivity analyses were done to investigate the effect of HIV clinical variables and non-essential variables, but with reduced power.

Statistical analyses were performed using XLSTAT version 2019.1.1.

## Supplementary Material

Supplementary Methods

Supplementary Figures

Supplementary Table 1
