# Supplementary material for: Inverse relationship between leukocyte telomere length attrition and blood mitochondrial DNA content loss over time
Source: Aging (Albany NY). 2020 Jul 23;12(15):15196–221. doi: 10.18632/aging.103703 (PMC7467389; doi:10.18632/aging.103703)
Supplement: Supplementary Methods [file aging-12-103703-s001..pdf]

SUPPLEMENTARY METHODS

Study sample

HIV+ participants were enrolled in the CARMA cohort during routine clinical visits at four locations across Canada: British Columbia Women's Hospital in Vancouver, British Columbia; the Centre Hospitalier Universitaire Sainte-Justine in Montreal, Quebec; the Hospital for Sick Children in Toronto, Ontario; and the Children's Hospital of Eastern Ontario in Ottawa, Ontario. CARMA study participant visits occurred annually between 2008-2013, and every 2-3 years thereafter. The majority (293/300) of the HIV-uninfected participants were invited to participate through word of mouth and advertisements placed in regions of Vancouver that have higher representation of at-risk individuals. The remaining 7 were born from HIV+ mothers previously enrolled in CARMA and are HIV-exposed uninfected (HEU) participants.

Demographic and clinical data

Demographic data were self-reported at study entry for all participants, and substance use data were self-reported during study visits for participants ≥14 years old. HIV clinical information were collected from medical records when available. Complete cART history was documented, from which cART status at visit was derived. HCV and HBV infection history were self-reported and confirmed by medical records when available. Missing data from essential variables were imputed from the nearest visit with available data, except for detectable/undetectable HIV pVL, which was imputed from on/off ART at visit, and vice versa. Of the 8 variables that required imputation, an average of 1.5% of the data were imputed ranging from 0.3% to 2.4%.

qPCR

For both assays, quality control was applied as previously described [1, 2]. Briefly, individual assays were accepted or rejected based on the measurements of two internal controls, the amplification efficiency of both amplicons as calculated using a standard curve, the average variability between all technical duplicates in the plate, and a negative control. Measurements of individual specimens were accepted if they fell within the linear range of the assay and if the difference between technical duplicates was <15%. Measurements that were not accepted were repeated at most once more and subjected to the same QC criteria. All qPCR data assayed for this study passed QC.

Statistical analysis

In cross-sectional models involving all participants, potential explanatory variables first included age, ethnicity, tobacco smoking, cannabis use, alcohol use, opioid use, HIV status, and HCV infection history. In models restricted to HIV+ participants only, the above variables were considered in addition to CD4 count and detectable HIV pVL. LTL was also considered in all WB mtDNA content models and vice versa. After the final model was developed, the following non-essential variables were considered in sensitivity analyses with reduced power: household income, highest education level completed, HCV detectable pVL, HIV peak pVL, CD4 nadir, ART-naïve status, and third drug in the cART regimen. HBV infection history was considered only in HIV+ analyses, as only 2 HIV-uninfected participants were ever infected with HBV. Longitudinal models included LTL and WB mtDNA content at first visit as well as changes in HIV detectable pVL and on/off ART as additional potential explanatory variables. The subgroup model of ΔLTL/year among HIV-uninfected participants considered only continuous variables (age, baseline LTL, and ΔWB mtDNA content/year) because of reduced sample size.

REFERENCES

1. Hsieh AYY, Saberi S, Ajaykumar A, Hukezalie K, Gadawski I, Sattha B, Côté HCF. Optimization of a Relative Telomere Length Assay by Monochromatic Multiplex Real-Time Quantitative PCR on the LightCycler 480: Sources of Variability and Quality Control Considerations. J Mol Diagn. 2016; 18:425–437. https://doi.org/10.1016/j.jmoldx.2016.01.004 PMID:26972047

2. Hsieh AYY, Budd M, Deng D, Gadawska I, Côté HCF. A Monochrome Multiplex Real-Time Quantitative PCR Assay for the Measurement of Mitochondrial DNA Content. J Mol Diagn. 2018; 20:612–620. https://doi.org/10.1016/j.jmoldx.2018.05.001 PMID:29936256
