# Supplementary material for: Inverse relationship between leukocyte telomere length attrition and blood mitochondrial DNA content loss over time
Source: Aging (Albany NY). 2020 Jul 23;12(15):15196–221. doi: 10.18632/aging.103703 (PMC7467389; doi:10.18632/aging.103703)
Supplement: Supplementary Figures [file aging-12-103703-s003..pdf]

# SUPPLEMENTARY FIGURES

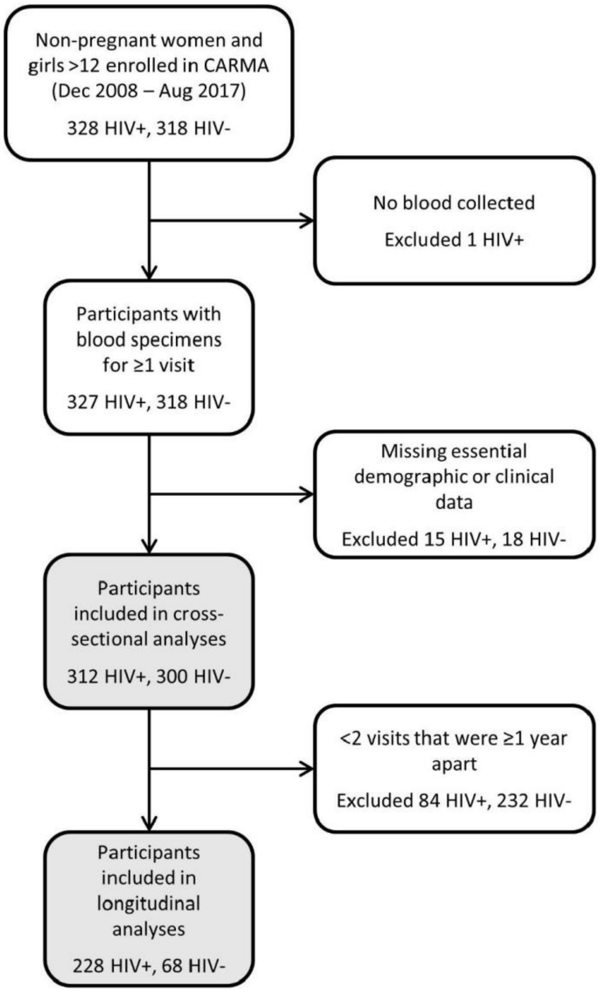


**Supplementary Figure 1. Study selection schematic. Inclusion and exclusion criteria for present study are described.** Required demographic or clinical data include age, ethnicity, opioid use, cigarette smoking, cannabis use, alcohol use, HIV status, HIV viral load, CD4 count, and HCV infection ever. Shaded boxes indicate samples for analysis.

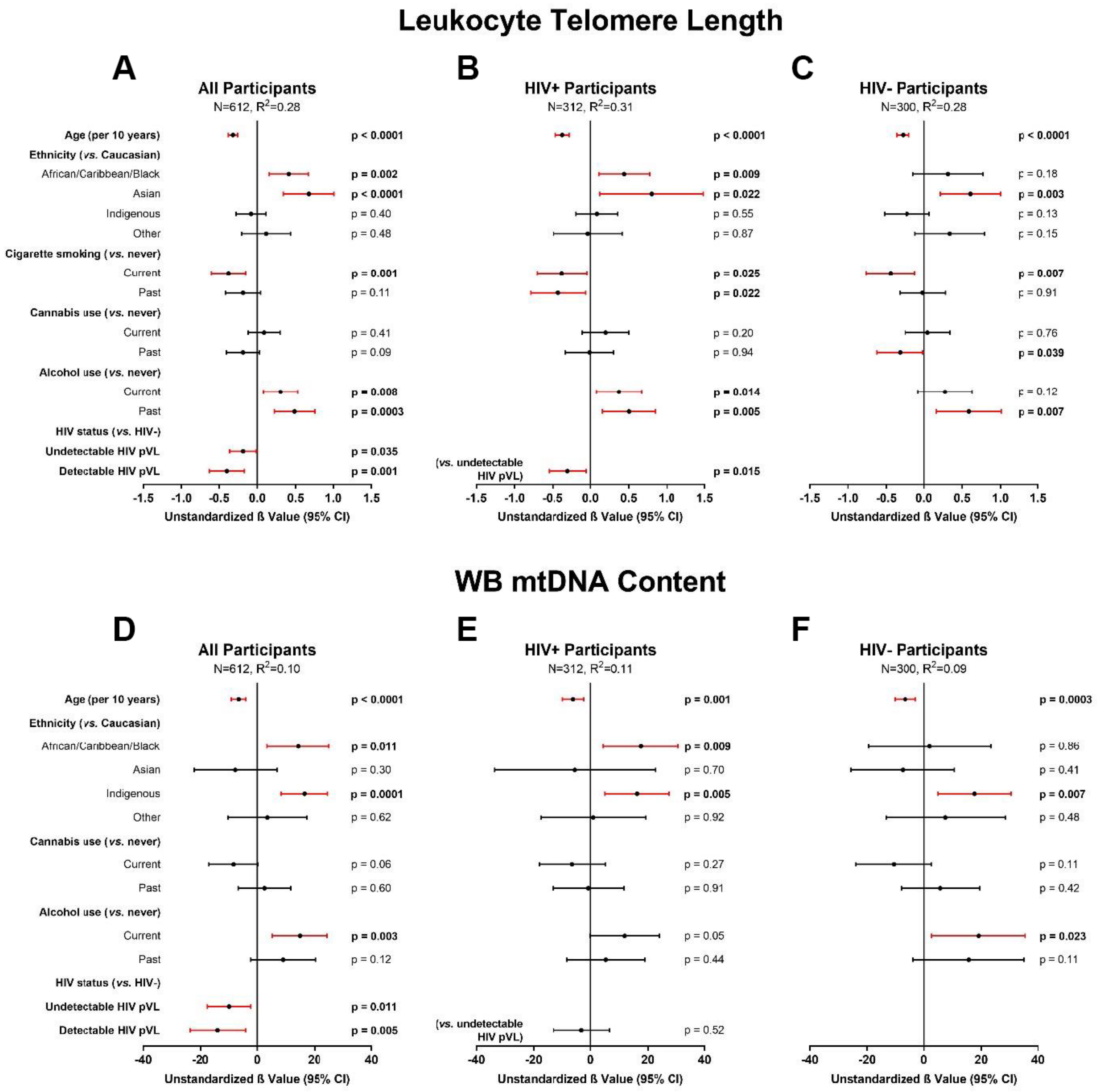


**Supplementary Figure 2. Multivariable modelling of cross-sectional leukocyte telomere length (LTL) and whole blood mitochondrial DNA (WB mtDNA) content with unstandardized effect sizes (β).** Final selected multivariable linear regression models of cross-sectional LTL (variance inflation factor (VIF)≤2.1) in (**A**) all, (**B**) HIV+, and (**C**) HIV- participants, and WB mtDNA content (VIF≤1.3) in (**D**) all, (**E**) HIV+, and (**F**) HIV- participants. Final models among all participants were selected automatically by minimizing Akaike's Information Criterion. Statistical significance depicted by red confidence intervals; negative unstandardized β values indicate associations with either shorter LTL or lower WB mtDNA content and vice versa. Coefficients of determination ($R^2$) are shown for each model.

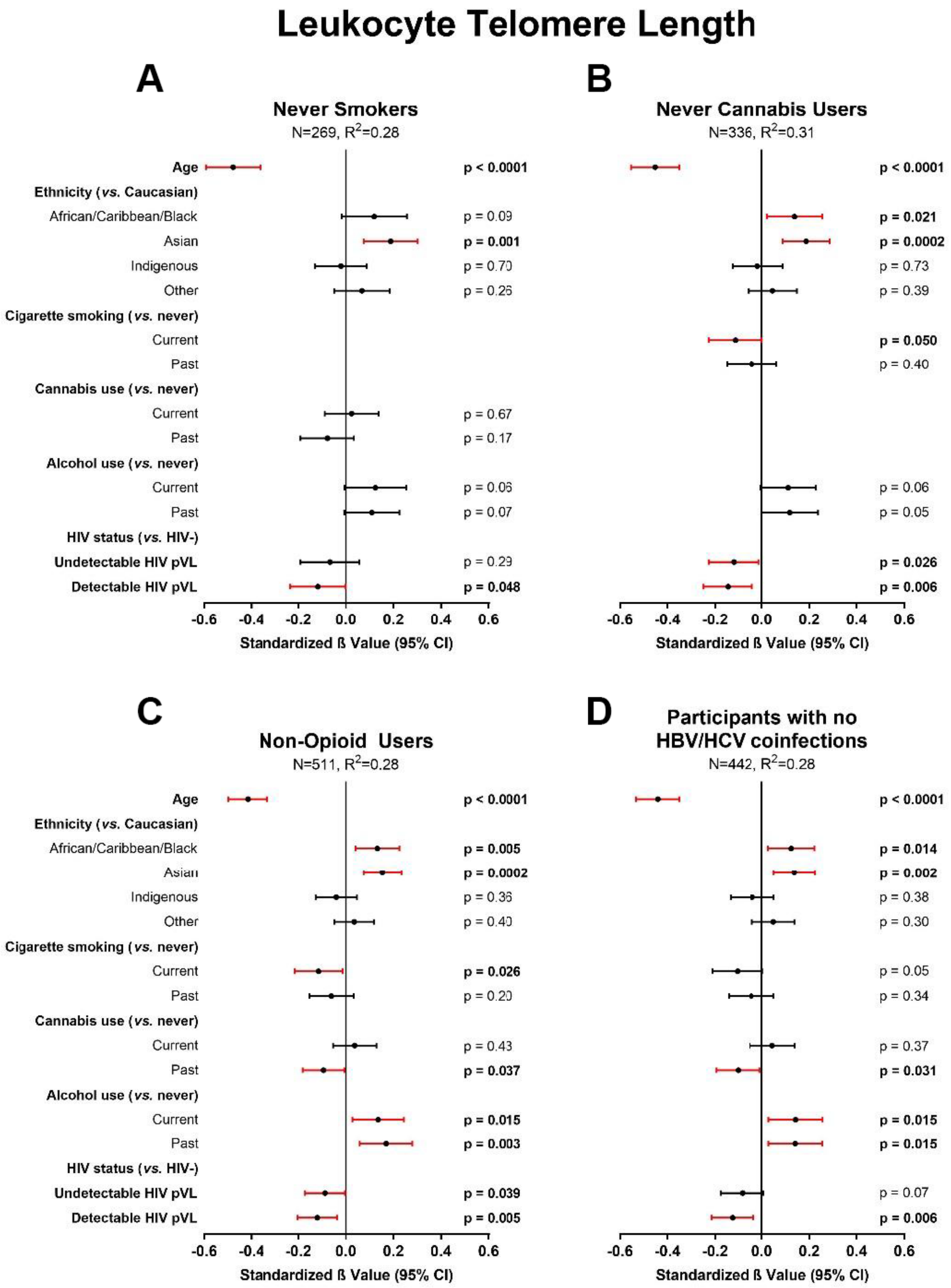


**Supplementary Figure 3. Subgroup analyses of cross-sectional leukocyte telomere length (LTL).** Multivariable subgroup analyses of LTL (variance inflation factor (VIF) ≤1.7) among (**A**) never smokers, (**B**) never cannabis users, (**C**) non-opioid users, and (**D**) participants with no history of HBV or HCV coinfection. Statistical significance depicted by red confidence intervals; negative standardized β values indicate associations with shorter LTL and vice versa. Coefficients of determination ($R^2$) are shown for each model.

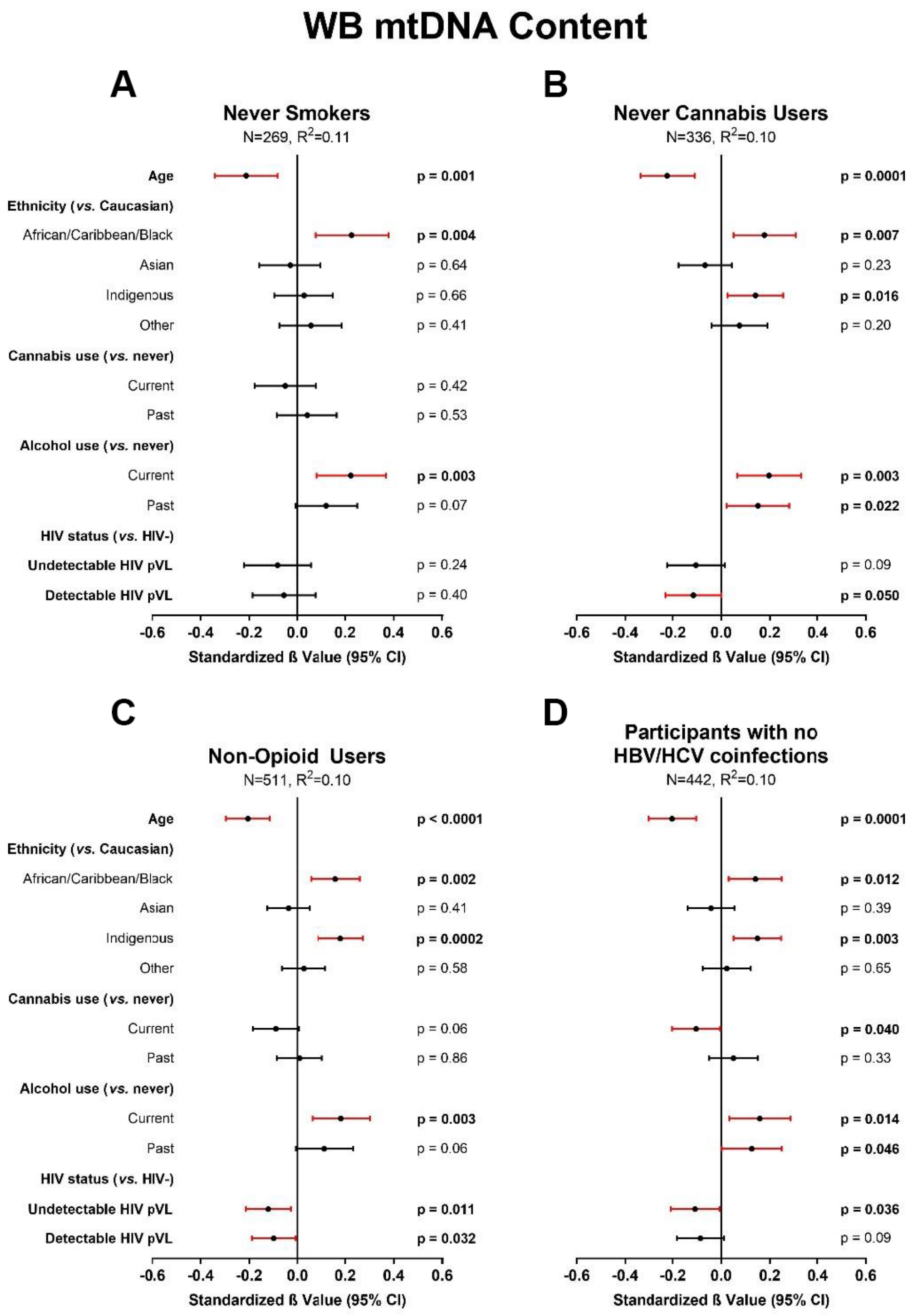


**Supplementary Figure 4. Subgroup analyses of cross-sectional whole blood mitochondrial DNA (WB mtDNA) content.** Multivariable subgroup analyses of WB mtDNA content (variance inflation factor (VIF) ≤1.4) among (**A**) never smokers, (**B**) never cannabis users, (**C**) non-opioid users, and (**D**) participants with no history of HBV or HCV coinfection. Statistical significance depicted by red confidence intervals; negative standardized β values indicate associations with lower WB mtDNA content and vice versa. Coefficients of determination ($R^2$) are shown for each model.

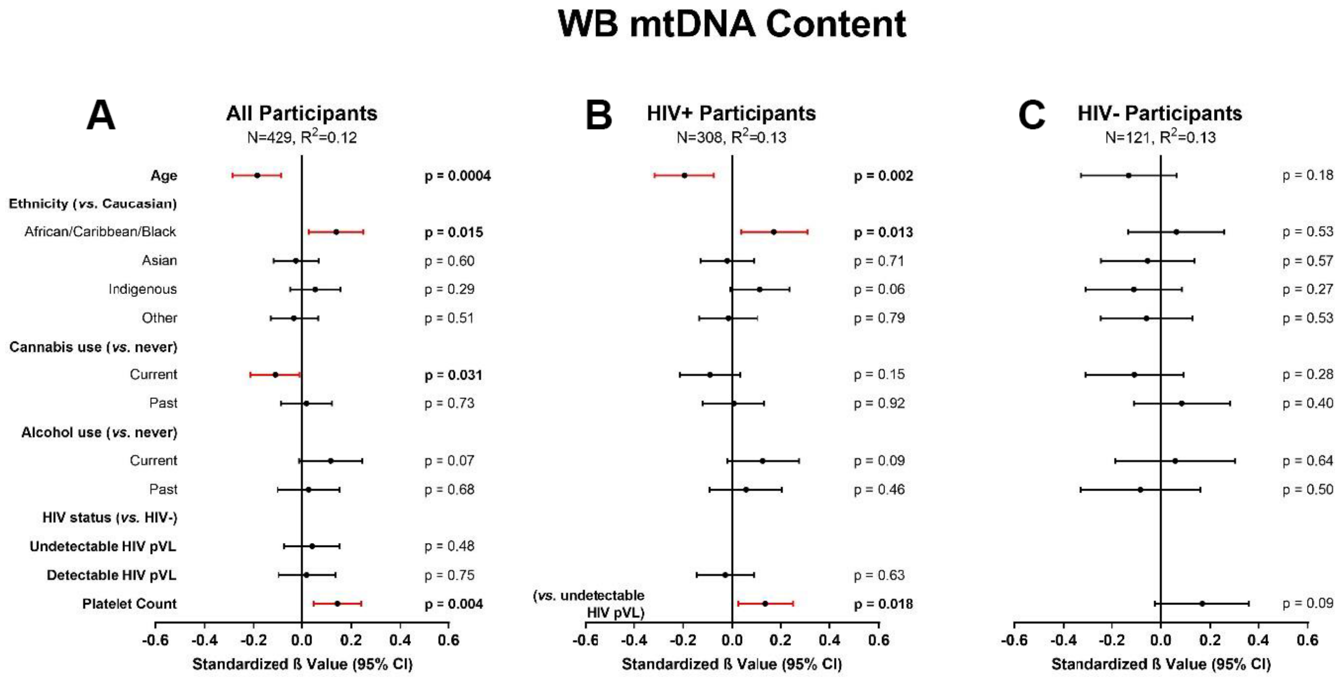


**Supplementary Figure 5. Sensitivity analyses of cross-sectional whole blood mitochondrial DNA (WB mtDNA) content with platelet count.** Multivariable sensitivity analyses of WB mtDNA content with platelet count (variance inflation factor (VIF) ≤1.3) among in (**A**) all (P<0.0001), (**B**) HIV+ (P<0.0001), and (**C**) HIV- (P<0.11) participants. Statistical significance depicted by red confidence intervals; negative standardized β values indicate associations with lower WB mtDNA content and vice versa. Coefficients of determination ($R^2$) are shown for each model.

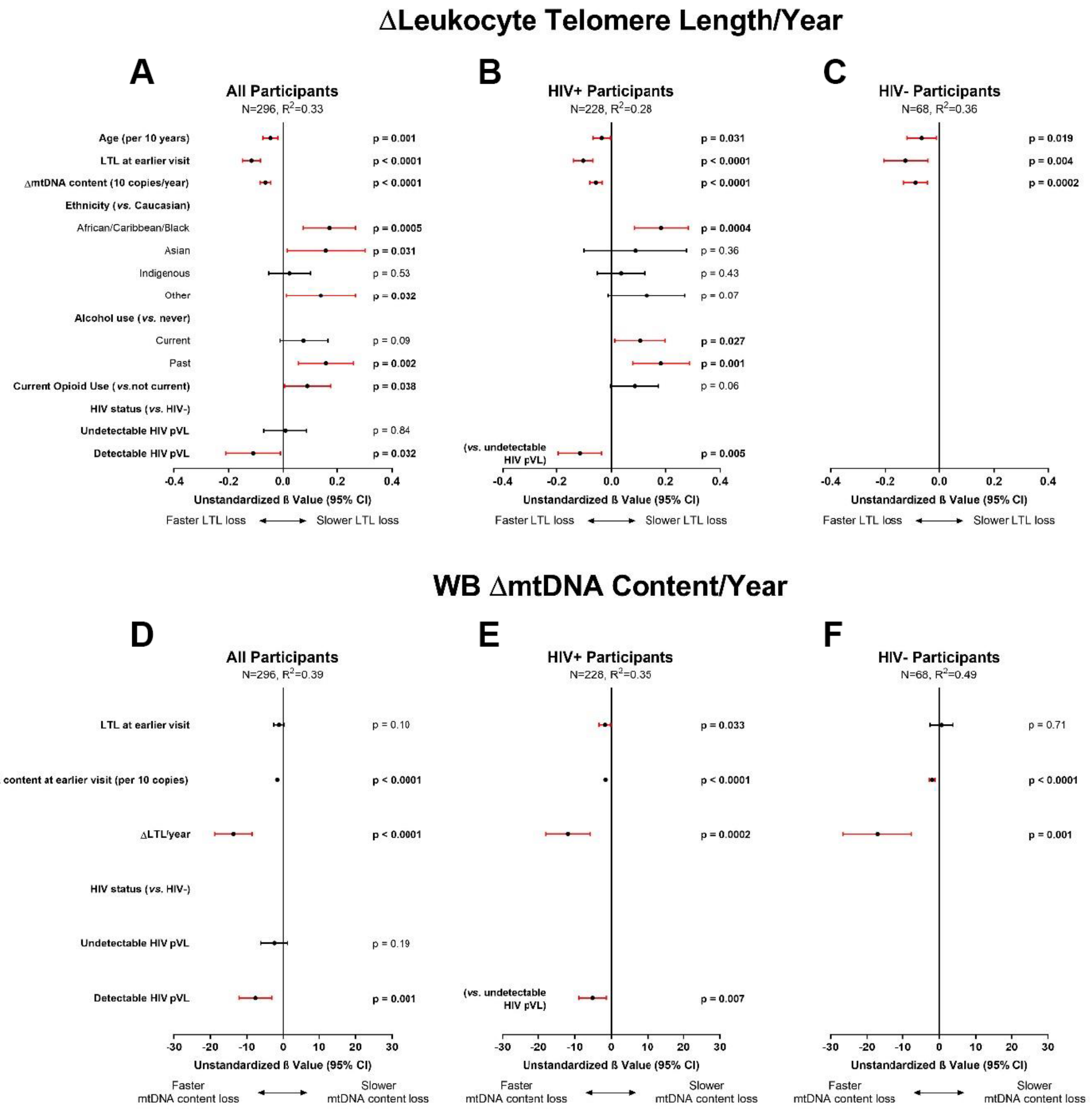


**Supplementary Figure 6. Multivariable modelling of longitudinal change in leukocyte telomere length (LTL) and whole blood mitochondrial DNA (WB mtDNA) content with unstandardized effect sizes (β).** Final selected multivariable linear regression models of longitudinal LTL (variance inflation factor (VIF) ≤1.5) in (**A**) all, (**B**) HIV+, and (**C**) HIV- participants, and WB mtDNA content (VIF≤1.2) in (**D**) all, (**E**) HIV+, and (**F**) HIV- participants. Final models among all participants were selected automatically by minimizing Akaike's Information Criterion (AIC). Statistical significance depicted by red confidence intervals; negative standardized β values indicate associations with either faster LTL loss or faster WB mtDNA content loss and vice versa. Coefficients of determination ($R^2$) are shown for each model.

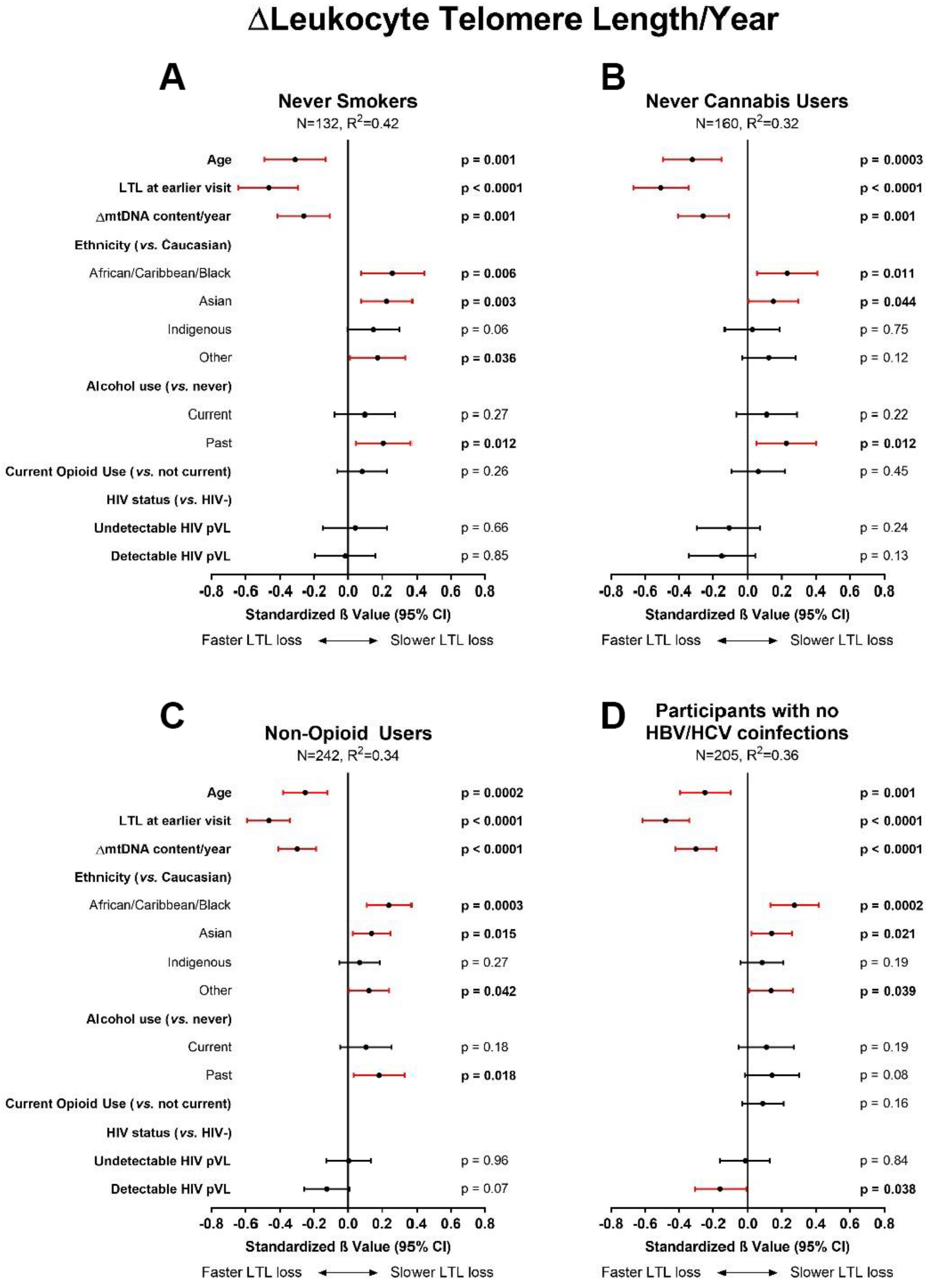


**Supplementary Figure 7. Subgroup analyses of longitudinal change in leukocyte telomere length (LTL).** Multivariable subgroup analyses of longitudinal ΔLTL/year (variance inflation factor (VIF) ≤1.7) among (**A**) never smokers, (**B**) never cannabis users, (**C**) non-opioid users, and (**D**) participants with no history of HBV or HCV coinfection. Statistical significance depicted by red confidence intervals; negative standardized β values indicate associations with faster LTL loss and vice versa. Coefficients of determination ($R^2$) are shown for each model.

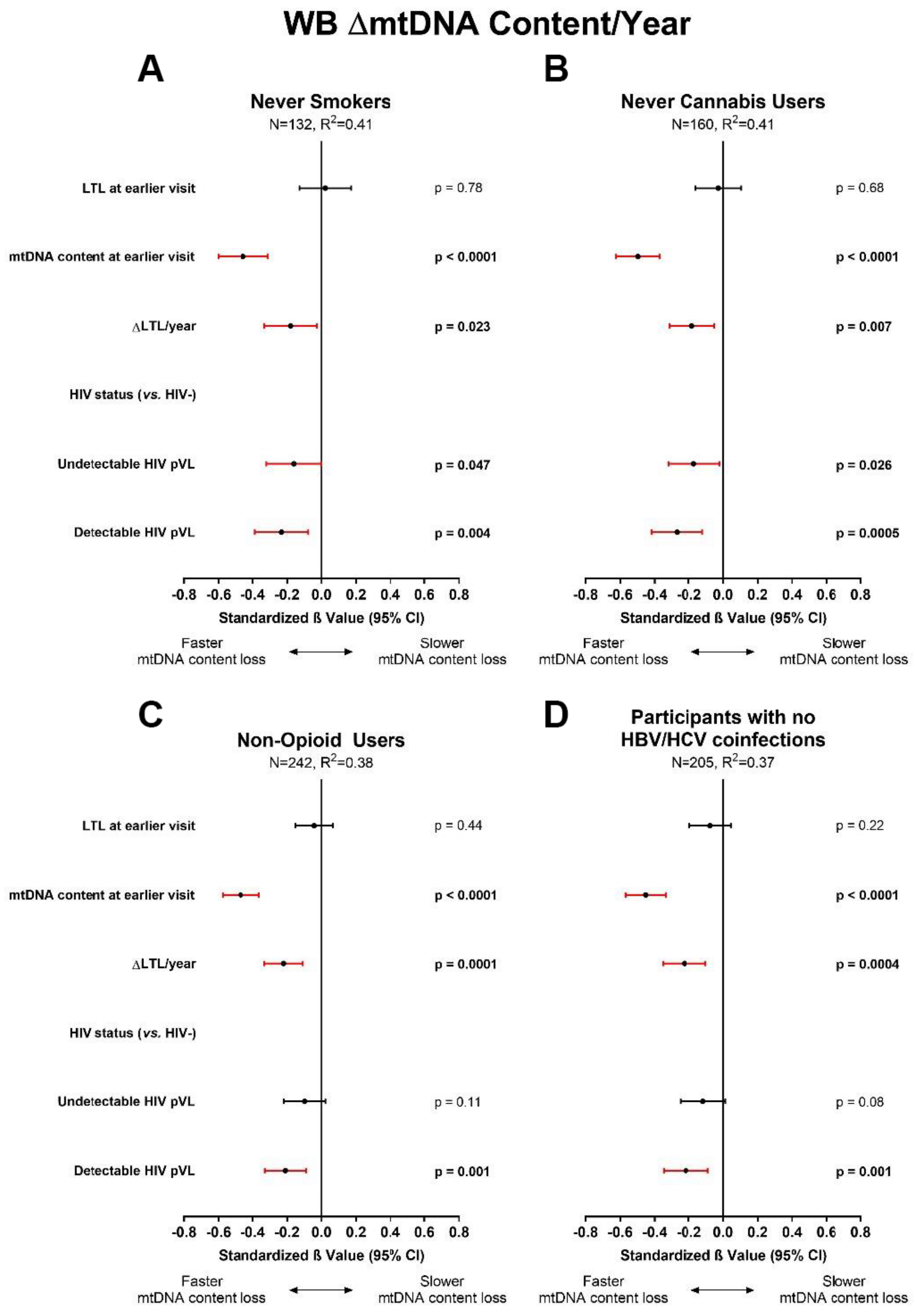


**Supplementary Figure 8. Subgroup analyses of longitudinal change in whole blood mitochondrial DNA (WB mtDNA) content.** Multivariable subgroup analyses of longitudinal WB ΔmtDNA content/year (variance inflation factor (VIF) ≤1.3) among (**A**) never smokers, (**B**) never cannabis users, (**C**) non-opioid users, and (**D**) participants with no history of HBV or HCV coinfection. Statistical significance depicted by red confidence intervals; negative standardized β values indicate associations with faster WB mtDNA content loss and vice versa. Coefficients of determination ($R^2$) are shown for each model.

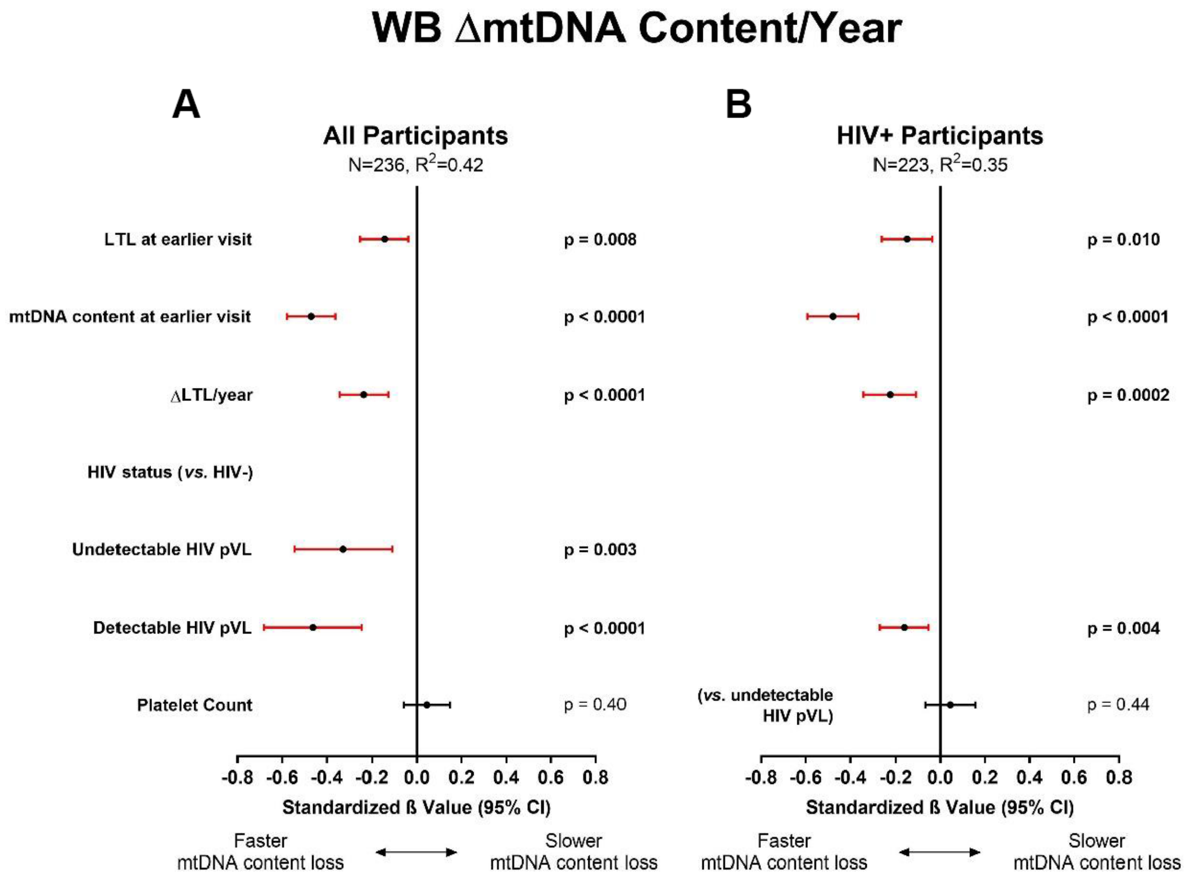


**Supplementary Figure 9. Sensitivity analyses of change in longitudinal whole blood mitochondrial DNA (WB mtDNA) content with platelet count.** Multivariable sensitivity analyses of WB ΔmtDNA content/year with platelet count (variance inflation factor (VIF) ≤1.2) among in (**A**) all and (**B**) HIV+ participants. Statistical significance depicted by red confidence intervals; negative standardized β values indicate associations with faster WB mtDNA content loss and vice versa. Coefficients of determination ($R^2$) are shown for each model.
