# Supplementary material for: Inverse relationship between leukocyte telomere length attrition and blood mitochondrial DNA content loss over time
Source: Aging (Albany NY). 2020 Jul 23;12(15):15196–221. doi: 10.18632/aging.103703 (PMC7467389; doi:10.18632/aging.103703)
Supplement: Supplementary Table 1 [file aging-12-103703-s002..pdf]

SUPPLEMENTARY TABLE

Supplementary Table 1. Essential and non-essential variables.

Essential variables	Non-Essential variables
Age	BMI
Ethnicity	Household Income
Tobacco Smoking	Highest Education
Alcohol Use	HIV Peak Viral Load
Cannabis Use	ART-naïve at visit
Opioid Use	Lowest CD4 Count Recorded
HIV Status	HBV Infection Ever
HIV Viral Load	HCV Active Infection
CD4 Count	Platelet Count
HCV Infection Ever	
